# In-vitro propagation, callus culture and bioactive lignan production in *Phyllanthus tenellus* Roxb: a new source of phyllanthin, hypophyllanthin and phyltetralin

**DOI:** 10.1038/s41598-020-67637-8

**Published:** 2020-06-30

**Authors:** Harichandra A. Nikule, Kirti M. Nitnaware, Mahadev R. Chambhare, Nitin S. Kadam, Mahesh Y. Borde, Tukaram D. Nikam

**Affiliations:** 1 Department of Botany, Savitribai Phule Pune University, Pune, 411 007 India; 2 Central Instrumentation Facility, Savitribai Phule Pune University, Pune, 411 007 India; 3 Department of Botany, Hutatma Rajguru Mahavidyalaya, Rajgurunagar Dist., Pune, 410 505 India; 4 Design Innovation Centre, Department of Chemistry, Savitribai Phule Pune University, Pune, 411 007 India

**Keywords:** Plant sciences, Plant biotechnology, Plant physiology

## Abstract

This is the first report on identification and quantification of important hepatoprotective and anticancer polyphenolic lignans such as phyllanthin (PH), hypophyllanthin (HPH), niranthin (NH) and phyltetralin (PT) in natural plant and in vitro cultures of *Phyllanthus tenellus* Roxb. The identification of lignans was carried out by Liquid Chromatography–High Resolution Mass Spectrometry (LC–HRMS) and quantified using High-Performance Liquid Chromatography (HPLC). In addition, an efficient protocol has been developed for multiple shoot induction in nodal explants of in vitro derived shoots of *P. tenellus*. Maximum number of shoot regeneration (7.83 ± 0.15) was achieved on medium incorporated with 1.0 mg/l 6-Benzylaminopurine (BAP). The medium containing Indole-3-acetic acid (IAA) 2 mg/l was superior for induction of rooting in in vitro raised shoots. The plantlets were acclimatized to the field condition with 100% survival. The quantitative HPLC analysis showed that the lignan content was variable with the auxins and cytokinins incorporated in the medium. The lignan content was higher in callus grown on Murashige and Skoog (MS) medium + 2.0 mg/l Naphthaleneacetic acid (NAA). The reported protocol can be used for mass propagation and application of biotechnological approaches for improvement of *P. tenellus*. The results indicate intriguing possibilities for the utilization of *P. tenellus* plant parts as an alternative source and of callus culture to scale up bioactive lignan production for pharmaceutical applications.

## Introduction

*Phyllanthus tenellus* Roxb. (Long-stalk *Phyllanthus*, family- Phylanthaceae) is an annual, multipurpose medicinal herb*.* It has been used traditionally for urolithiasis in some part of the world, and therefore is also referred to as a stone breaker^1^. The herb has immunomodulatory, analgesic, anti-inflammatory, antimicrobial, antifungal, anti-hepatitis, antidiabetic and antitumor activity^1–3^. In addition, the plant extract is effective for curing the kidney, urinary bladder and intestinal disorders^4,5^. The phytochemical analysis showed the presence of medicinally important metabolites, such as niranthin, nirtetralin, hinoquinine and geranin^6–8^. Niranthin is highly effective as anti- hepatitis B surface antigen^7^. While nirtetralin and nirtetralin A, B effectively suppressed the secretion of the HBV (Hepatitis B virus); geraniin showed greater antioxidant and antihyperglycemic activities and niranthin has antiviral and anticancer activity^7,9^. Phyllanthin and hypophyllanthin are the most important bioactive lignans found in *Phyllanthus* species. Numerous studies have proved that phyllanthin and hypophyllanthin have cardioprotective, antihepatitis, antiviral, antifibrotic, anti-inflammatory, immunomodulatory, nephroprotective and anticancer activity^10,11^. To the best of our acquaintance, this is the first report on detection of medicinally important phyllanthin, hypophyllanthin and phyltetralin in *P. tenellus*.

There are some reports available on in vitro propagation of different species of *Phyllanthus* such as *P. urinaria, P. stipulatus* and *P. caroliniensis*^12–14^. Despite having significant medicinal importance, *Phyllanthus tenellus* has so far received very meagre consideration on in vitro propagation and metabolite production^15^. Plant tissue culture has importance in propagation and production of secondary metabolites from plants that are economically and medicinally important and rare. This technique also delivers a significant approach for the enhanced production of plant metabolites by changing applications and combinations of growth regulators and application of elicitors in the culture media^16,17^.

Therefore, in the present study, attempts were made to develop an improved in vitro propagation protocol in *P. tenellus*. Study was also done to detect and test the ability of biosynthesis in biomass raised in plant tissue culture and natural environment for bioactive lignans phyllanthin, hypophyllanthin, niranthin and phyltetralin by using LC-HRMS and HPLC technique.

## Materials and methods

### Establishment of in vitro shoot cultures

The six-week-old *Phyllanthus tenellus* Roxb. plants grown naturally in Botanical garden, Savitribai Phule Pune University, Pune, India (Latitude 18.554499, Longitude 73.825729) were used as a source of explants. The plant herbarium was submitted and authenticated at Botanical Survey of India, Western Circle. Pune, India (the specimen Voucher No. is BSI/WRC/Iden./2015/427, TDN-6). The nodal (1 cm in length with one node) and leaf explants (3 × 3 mm size) were washed six times with sterilized distilled water and disinfected by using HgCl_2_ (0.1% w/v) for 3.5 min. After washing with sterilized distilled water for six times, the fresh cut was applied at the exposed cut end of the explants. For establishment of shoot cultures, the explants were inoculated on medium^18^ containing BAP (1 mg/l). The explants were also inoculated on plant growth regulators free MS medium and treated as a control. The root, nodal and leaf explants excised from in vitro cultures were used in experiments on shoot regeneration on medium containing BAP, 6-furfurylaminopurine (Kin), Thidiazuron (TDZ), IAA, Indole-3-butyric acid (IBA), NAA and 2,4-Dichlorophenoxyacetic acid (2,4-D) alone or in combination. Using nodal explants from in vitro raised cultures, the maintenance of multiple shoot regeneration was carried out on media fortified with 1.0 mg/l of BAP. All the in vitro cultures were grown in culture room with arrangement of 8 h fluorescent light (about 40 μmol m^−2^ s^−1^) and 16 h dark period and 25 ± 2 °C temperature.

### In vitro rooting and acclimatization

The individual shoots with one or two nodes were transferred for rooting on medium fortified with NAA and IAA (0.0 to 3.0 mg/l). After removal of agar medium, well grown plantlets were transferred to pots consisting of mixture of garden soil and sand (3:1). Initially for two weeks every day in the morning and evening, water was applied to the plantlets and acclimatized under shade-net (50% natural light cut). Then plantlets were transferred to field condition (Temperature in the range of 12 °C to 38 °C).

### Induction, proliferation and maintenance of callus

For callus induction and proliferation, the nodal and leaf explants from in vitro raised shoots were used. This experiment was carried out on a medium containing BAP, Kin, TDZ, 2, 4-D and NAA (00 to 2.0 mg/l) separately and 2, 4-D (2.0 mg/l) or NAA (2.0 mg/l) together with BAP, Kin or TDZ (00 to 3 mg/l). The callus was maintained by subculturing 300 mg of healthy callus onto fresh callus proliferation media (MS + 2.0 mg/l NAA and MS + 2.0 mg/l 2, 4-D) at an interval of four weeks (18 subcultures). The fresh weight of callus was determined after four weeks of growth. To obtain dry weights, the calli were dried at 60 °C in a hot air oven until constant weight (72 h).

### Extraction of lignans and standard sample preparation

The dried biomass obtained from different experiments were ground to powder using mortar and pestle. On some modification, the method elaborated by Nitnaware et al.^19^ was used for extraction. The sample powder (0.5 g) was suspended in acetone (20 ml) and ultra-sonicated for 45 min at ambient conditions. Then the suspension was filtered through Whatmann filter paper No.1 and the residue was extracted again in acetone (20 ml) three times. The filtrate was pooled together and evaporated using rotary evaporator at 56 °C. The resultant residue was liquefied in HPLC grade methanol. The mixtures were filtered through 0.22 µm filter and used for HPLC and LC-HRMS analysis.

The standard lignan compounds PH, HPH, NH and PT were purchased from Genetix Biotech Asia Pvt. Ltd., New Delhi, India. The solution of standard compounds (1 mg in 1 ml of HPLC grade methanol) was prepared separately. The individual solution and the mixture of a solution of the standard compounds were diluted to make levels of 0.2, 0.4, 0.6, 0.8 and 1.0 mg/ml. Each solution was filtered through 0.22 µm syringe filter and used for HPLC analysis.

The specificity of lignan compounds extracted from calli subjected to various treatments was determined by comparing the retention time (*Rt*) and molecular ions (m/z) with respective standard compounds after chromatographic separation. The quantity of lignans in the plant sample was determined from the linear regression equation of the standard calibration curve.

### HPLC of standard compounds and extracts

The lignans PH, HPH, NH, and PT were assayed by HPLC (Agilent 1260 Infinity II with Diode Array Detector). A BDS Hypersil C_18_ column (250 × 4.6 mm) at 25 °C was used for separation of compounds. HPLC parameters were applied by referring Murugaiyah and Chan method^20^ with some modifications. The chromatographic separation was carried out using flow rate of 0.5 ml/min of acetonitrile: water (55:45, v/v) isocratic mobile phase for 45 min. 10 µl of sample volume was used for injection and the detector was operated at 254 nm.

### Liquid chromatography high performance mass spectrometry analysis

The sample analysis was carried out using Thermo Fisher Scientific LC system and Bruker made Impact HD mass spectrometer interfaced with electrospray ionization (ESI) and time-of-flight (TOF). The C_8_ column (Dionex Bonded Silica, 250 mm × 4.6 mm, 5 µm) was used for separation at 25 °C. The acidification of water and acetonitrile was carried out by adding formic acid (0.1% v/v). The acidified mobile phase of water and acetonitrile in a ratio of 40:60 (v/v) was used in isocratic mode. The volume of 15 µl sample and standard mixture was injected separately. The flow rate was set at 0.7 ml/min for 35 min. The detection of components of mixture was carried out using diode array detector at 190–400 nm. The positive ESI mode of mass spectrometer was used for ionization. Nitrogen gas was used as nebulizing and drying gas at the flow rate of 7 l/min. The spectra were documented in the mass range of m/z 50–1,200. The system capillary parameters were adjusted to temperature 200 °C, nebulizer pressure at 1.7 bars, capillary voltage (VCap) 4,500 V, and charging voltages 2000 V. The instrument was controlled through software like chromeleon for LC, otofcontrol (3.4 version) for MS and Hystar was used for LC–MS. The exact mass of the molecular ions were assigned through Compass Data Analysis software (Version 4.2, Bruker, USA).

### Statistical analysis

In the present study, experiments were performed in a completely randomized design with at least fourteen replicates. Each experiment was repeated at least thrice and data were recorded after 28 days of culture. The data was analyzed by one-way ANOVA using Duncan’s Multiple Range Test (*P* ≤ 0.5 levels).

## Results and discussion

### Shoot induction and multiplication

**Figure 1:**
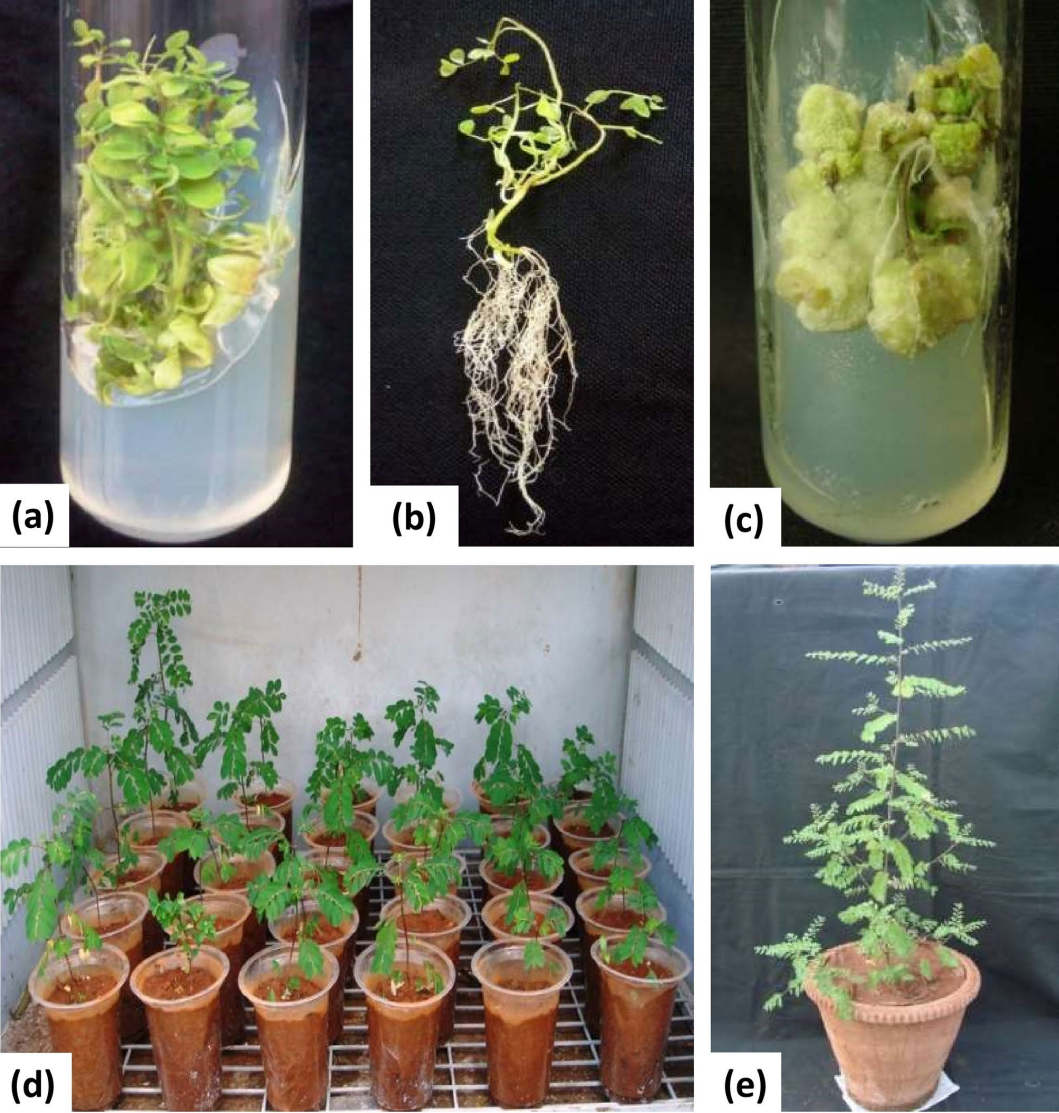
In vitro propagation of *P. tenellus*: (**a**) Multiple shoots formation in nodal explant on MS + 1 mg/l BAP, (**b**) Root formation in nodal explant on MS + 2 mg/l IAA, (**c**) Callus induction and proliferation in leaf explant on MS + 2 mg/l 2, 4-D, (**d**) Hardened plantlets after 15 days, and (**e**) Well acclimatized plantlets after six months.

It is understood that the, culture medium, source and age of explants, surface sterilization treatments, microbial contaminants and environmental factors are the critical factors for establishment of in vitro cultures. Literature survey reveals that the choice of explants and growth regulators is a fundamental requirement for any plant regeneration protocol^21^. In the current study, the preliminary experiments were performed for establishment of in vitro shoot cultures using nodal and leaf explants obtained from field grown plants. The nodal explants (44%) showed positive response by 1–2 shoot regeneration on medium containing 1.0 mg/l BAP. While remaining nodal explants and all leaf explants did not show any response for shoot regeneration. To prevent or to minimize the chances of somaclonal variation, nodal explants are most preferred, as pre-existing meristem permits multiple shoot formation without the intervention of callus formation^22^. To optimize in vitro propagation protocol, in vitro regeneration of shoots from different parts of in vitro grown plantlets has received attention^23,24^. In the current study, in vitro produced nodal explants were found to be the best explants for maximum direct multiple shoot formation and elongation of shoots. These results were in accordance with the findings on in vitro shoot formation in *Phyllanthus urinaria*^14,25^ and *Phyllanthus amarus*^24^. However, the shoot regeneration percentage was affected by the type and concentrations of Plant Growth Regulators (PGRs). The shoot proliferation percent increased with elevated concentrations of BAP. On medium, incorporation of 1 mg/l of BAP alone, maximum number of explants responded for shoot regeneration (97.36 ± 0.46%) and produced maximum average number of shoots per explants (7.8 ± 0.15) (Fig. 1a). Comparatively, the shoot multiplication frequency was relatively low on MS medium containing Kin or TDZ (Table 1). The gradual decline in shoot formation response and increase in callus formation was found at higher concentrations of BAP, Kin and TDZ. The results of the current study confirm that, the cytokinin BAP was superior for induction of multiple shoot regeneration in nodal explants of *Phyllanthus* species such as *P. urinaria*^25^, *P. niruri*^26^, *P. amarus*^27^, *P. maderaspatensis*^28^ and *P. fraternus*^29^. However, contrasting results are also reported in *Phyllanthus amarus*^19,30^ and *Phyllanthus urinaria*^14^; where the efficiency of shoot proliferation on Kin and TDZ was superior to BAP.

**Table 1:** Effect of different concentrations of cytokinins on shoot regeneration in *Phyllanthus tenellus* Roxb.

PGRs	Conc. (mg/l)	Explants				
		Node				Leaf
		Explants responding for shoot regeneration %	No. of shoots ± SE	Shoot length ± SE (cm)	Morphological response	
Control	0.0	79.4 ± 0.44^i^	1.3 ± 0.12^j^	2.3 ± 0.12^a^	R, S	R, S
BAP	0.5	82.8 ± 0.40^h^	6.0 ± 0.12^b^	1.6 ± 0.12^cde^	S	N
	1	97.3 ± 0.46^a^	7.8 ± 0.15^a^	1.5 ± 0.09^def^	S	N
	2	91.2 ± 0.60^d^	4.5 ± 0.20^c^	1.5 ± 0.15^def^	S*	N
	3	88.2 ± 0.55^fg^	3.3 ± 0.15^e^	1.0 ± 0.15^h^	S*	*
Kin	0.5	91.9 ± 0.44^bcde^	1.6 ± 0.06^hi^	1.9 ± 0.03^b^	S	*
	1	93.3 ± 0.65^bcde^	1.6 ± 0.01^hi^	1.8 ± 0.09^bc^	S	*
	2	89.5 ± 0.87^ef^	1.7 ± 0.01^h^	1.7 ± 0.06^cd^	S	*
	3	85.8 ± 1.47^g^	1.6 ± 0.01^hi^	1.3 ± 0.06^fg^	S*	*
TDZ	0.5	77.2 ± 0.61^i^	1.1 ± 0.03^jk^	0.5 ± 0.06^ij^	S*	*
	1	77.2 ± 0.60^i^	1.0 ± 0.06^k^	0.4 ± 0.03^ijk^	S*	**
	2	74.2 ± 0.47^j^	1.0 ± 0.00^k^	0.3 ± 0.01^ijk^	S*	**
	3	71.1 ± 0.61^k^	0.9 ± 0.00^k^	0.2 ± 0.00^k^	S*	**

The value represents the means of three replicates with standard error (SE). Values sharing the different alphabets are statistically different from each other at P < 0.05.

*R* root, *S* shoot, *N* no change.

*Callus induction.

**Callus induction and proliferation.

The response of shoot regeneration in nodal explants declined on incorporation of auxins together with BAP. Similar results were recorded in *P. caroliniensis*^12^, *P. fraternus*^31^ and in *P. reticulatus*^32^. It is understood that the inclusion of auxins counteracts the effect of BAP for shoot formation. However, contrasting results were reported in *P. tenellus*^15^. It was suggested that only 3.5 shoots regeneration per nodal explants derived from seedlings was possible using optimized medium MS + Kin (0.8 mg/l) + Gibberellic acid (GA_3_) (0.3 mg/l) + IBA (0.2 mg/l). This difference might be possible due to differences in source of explants, the medium enriched with IAA, IBA, GA_3_, and Kin and lacking the use of BAP. In the present study, on incorporation of cytokinins and auxins alone and combination, it was observed that BAP alone was best for maximum number of shoots regeneration per nodal explants (7.8 ± 0.15) (Table 1). The variation in number of shoot formation might be attributed to the different cytokinins, their concentration, combination with other PGRs and source of explants.

### In vitro rooting of shoots and acclimatization

Inadequate rooting is a major constraint to the survival rate of plantlets in field conditions and the success of in vitro regeneration protocol^22^. In vitro raised shoots (3–4 cm long) were removed from the culture vessel and inoculated individually on medium with various concentrations of auxins such as IAA, IBA, and NAA (0.5, 1, 2 and 3 mg/l of each). The root formation percentage from the shoots was varied with the type and concentration of auxins are shown in Supplementary Table 1. The root formation was observed from the cut end and nodal portion of the shoots within 14 days, which form an extensive well-developed root system after 21 days of culture. The maximum root formation (100%) and number of roots per shoot were observed on medium containing 1–2 mg/l IAA (Fig. 1b, Supplementary Table 1). The incorporation of higher concentration of IAA (3 mg/l) or NAA or IBA (2.0–3.0 mg/l) induces callus intervening root formation. This difference in response may be attributed to the interaction of exogenous and endogenous levels of auxin in the medium and cultured shoots respectively as well as their uptake, transport and metabolism^22^. Similar observation was recorded in *Phyllanthus amarus*^19^ and *Phyllanthus maderaspatensis*^28^. On the contrary, the effectiveness of IBA over other auxins in rooting has been reported in *P. urinaria*^25^, *P. fraternus*^29^, *P. reticulatus*^32^ and in *P. tenellus*^15^.

The four-week-old well rooted plantlets (8–10 cm in height) showed good growth on transfer to pots containing garden soil and sand (3:1) and placed in shade-net house (50% light cut). After four weeks of hardening, all plantlets survived on transfer to field conditions. The plantlets produced normal flowers and fruits, phenotypically similar to the source plants (Fig. 1e).

### Callus induction and growth

Results of the experiments on callus induction and growth of callus are depicted in Table 2 and Supplementary Table 2. Among various permutations and combinations of PGRs, extensive callus formation was observed on medium containing 2–3 mg/l of individual auxins (2, 4-D, NAA, IAA, IBA) and cytokinins (BAP, Kin and TDZ). The induction of calluses from the cut ends was observed within two weeks of culture and by the end of the fourth week, the entire surface of explants was covered with callus. Texture and color of calluses varied with the type and concentration of growth regulators (Table 2, Supplementary Table 2). There was no significant difference observed on the growth of callus on incorporation of auxins together with cytokinins. Of the auxins and cytokinins tested, the best callus growth in terms of dry weight was recorded on 2, 4-D containing medium, next it was observed on NAA, IAA, IBA, TDZ, BAP, and Kin respectively. The callus on subculture to similar fresh medium at the interval of 28 days showed proliferation and growth over a period of 18 months. The callus produced on NAA, IAA, and IBA were compact, hard and whitish to gray-green in color. While on 2, 4-D, the callus was friable and slightly whitish to yellowish in color. The callus produced on Kin was whitish and compact while on BAP and TDZ, the callus was friable and greenish in color. Occasionally, rhizogenesis and shoot formation was observed in callus maintained on IAA or NAA and TDZ fortified medium respectively. However, no morphogenic response was observed on 2, 4-D incorporated media. Results of the current study revealed that 2, 4-D alone was superior to other PGRs for induction and proliferation of callus in both leaf and nodal explants of *Phyllanthus tenellus* Roxb. Similar effect of 2, 4-D on callus induction and proliferation was reported in *Phyllanthus urinaria*^14^, *Phyllanthus tenellus*^15^, *Phyllanthus debilis*^33^ and *Phyllanthus reticulatus*^32^.

**Table 2:** Effect of different concentrations of NAA alone and in combination with cytokinins on growth of callus in *Phyllanthus tenellus* Roxb.

PGRs	Conc. mg/l	Explants					
		Node			Leaf		
		Nature of callus	FW (g)	DW (mg)	Nature of callus	FW (g)	DW (mg)
Control	00	N	N	N	N	N	N
NAA	0.5	WGC	0.73 ± 0.6^c^	177.5 ± 3.1^c^	GWC	1.16 ± 0.3^c^	187.2 ± 2.3^c^
	1	WGC	1.12 ± 1.1^b^	201.4 ± 1.4^b^	GWC	1.81 ± 0.8^a^	232.6 ± 2.7^b^
	2	WGC	1.21 ± 1.2^a^	212.7 ± 3.3^a^	GWC	1.88 ± 0.7^b^	237.2 ± 1.8^a^
	3	WGC	1.22 ± 1.3^a^	204.8 ± 2.3^b^	GWC	1.75 ± 0.3^b^	228.6 ± 1.9^b^
NAA + BAP	0.5	GC	1.20 ± 1.1^a^	205.2 ± 1.3^b^	GC	1.25 ± 0.3^b^	225.3 ± 1.3^a^
	1	GC	1.00 ± 0.6^b^	195.6 ± 0.3^c^	GC	1.21 ± 0.6^a^	218.2 ± 1.1^b^
	2	GC	0.75 ± 0.4^c^	181.8 ± 0.8^d^	GC	0.86 ± 1.4^c^	199.5 ± 0.7^c^
	3	GC	0.71 ± 0.3^c^	175.3 ± 0.7^e^	GC	0.81 ± 1.7^c^	187.3 ± 1.2^d^
NAA + Kin	0.5	GWF	1.20 ± 0.3^a^	208.8 ± 5.5^a^	GWF	1.81 ± 0.3^c^	229.4 ± 5.2^a^
	1	GWF	0.95 ± 0.6^b^	189.8 ± 4.6^b^	GWF	1.68 ± 0.8^a^	212.4 ± 2.8^b^
	2	GWF	0.82 ± 0.8^c^	173.6 ± 4.1^c^	GWF	1.42 ± 0.7^b^	201.6 ± 4.3^c^
	3	GWF	0.76 ± 0.1^d^	162.5 ± 3.2^d^	GWF	1.35 ± 0.3^b^	188.7 ± 2.3^d^
NAA + TDZ	0.5	GC	1.15 ± 0.2^c^	201.4 ± 1.1^b^	GF	1.71 ± 0.2^d^	222.7 ± 5.6^b^
	1	GC	1.12 ± 0.3^a^	183.3 ± 2.8^a^	GF	1.62 ± 0.4^c^	204.6 ± 0.8^a^
	2	GC	1.00 ± 0.4^a^	163.7 ± 1.4^c^	GF	1.48 ± 1.2^a^	184.4 ± 4.1^c^
	3	GC	0.92 ± 0.2^b^	152.3 ± 3.6^d^	GF	1.23 ± 1.2^b^	161.3 ± 4.2^c^

The value represents the means of three replicates with standard error (SE). Values sharing the different alphabets are statistically different from each other at P < 0.05.

*N* no callus formation, *WGC* whitish green compact, *GC* green compact, *GWF* greenish white friable, *GWC* greenish white compact, *GF* green friable.

### Identification of bioactive lignans

**Figure 2:**
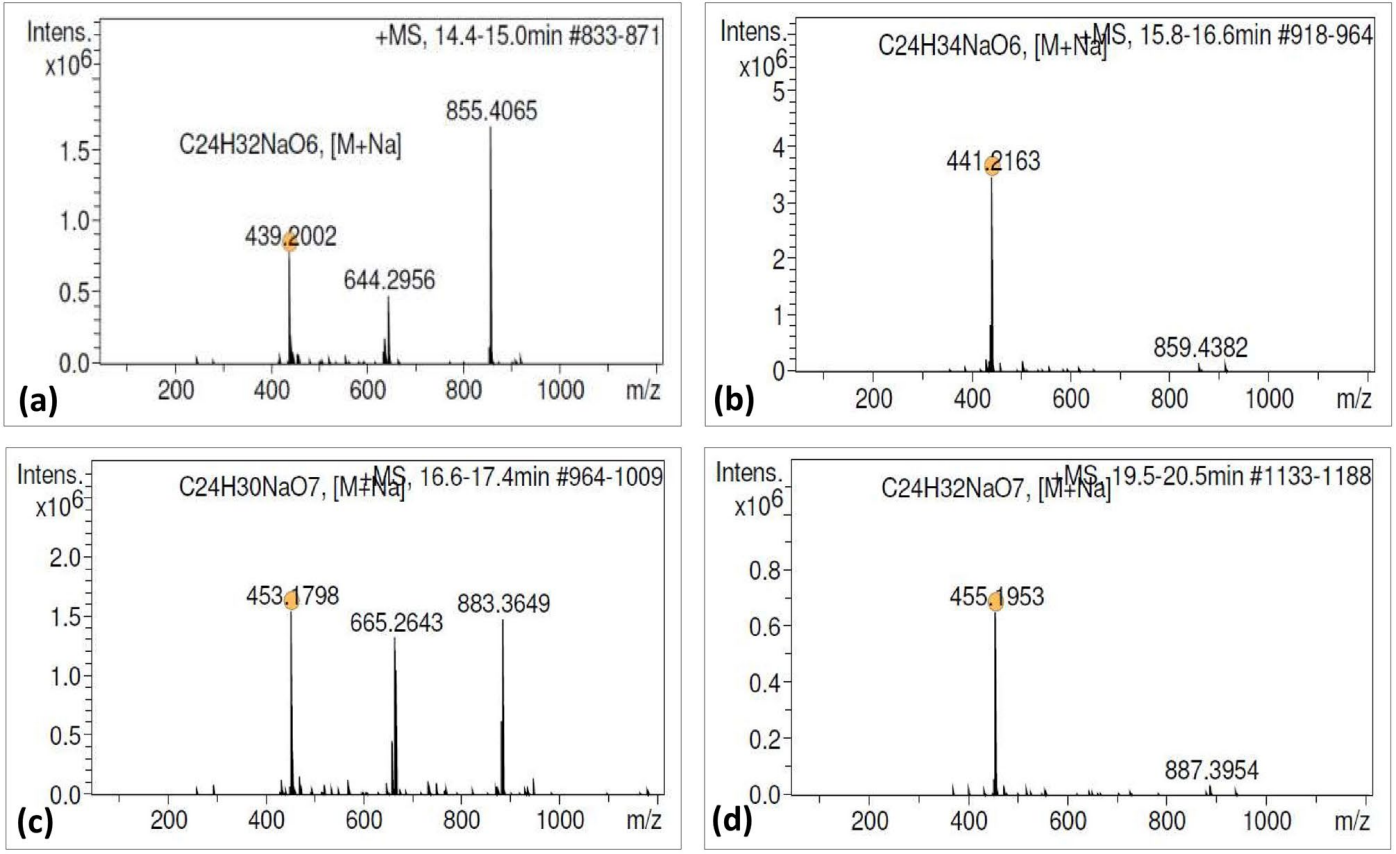
Product mass spectra of [M+Na] positive ions of reference standards: (**a**) Phyltetralin (m/z 439.2002), (**b**) Phyllanthin (m/z 441.2163), (**c**) Hypophyllanthin (m/z 453.1798), and (**d**) Niranthin (m/z 455.1953).

**Figure 3:**
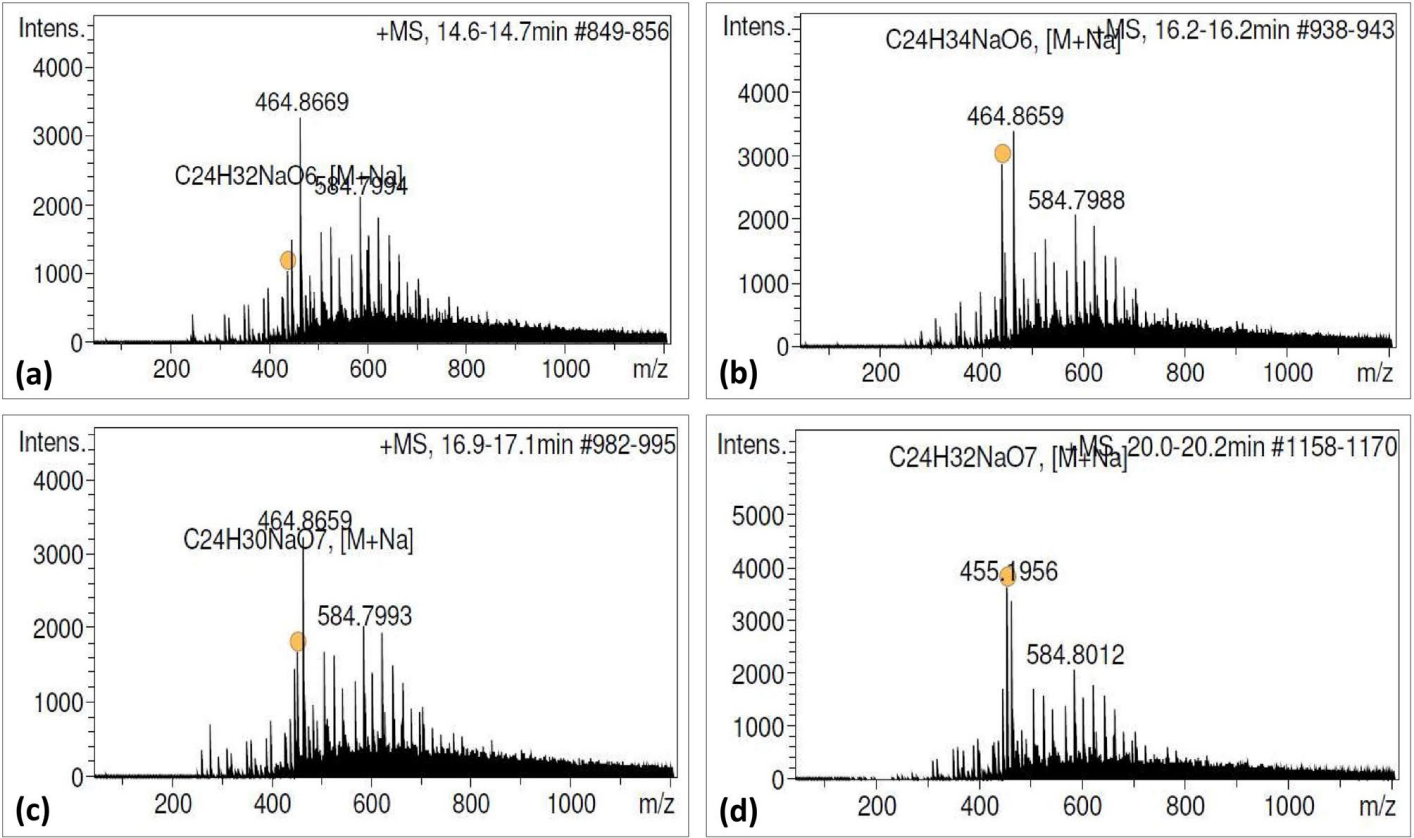
Product mass spectra of four lignans detected in *P. tenellus* by QTOF-MS in positive ion mode: (**a**) Phyltetralin (m/z 439.2007), (**b**) Phyllanthin (m/z 441.2173), (**c**) Hypophyllanthin (m/z 453.1806) and (**d**) Niranthin (m/z 455.1956).

**Figure 4:**
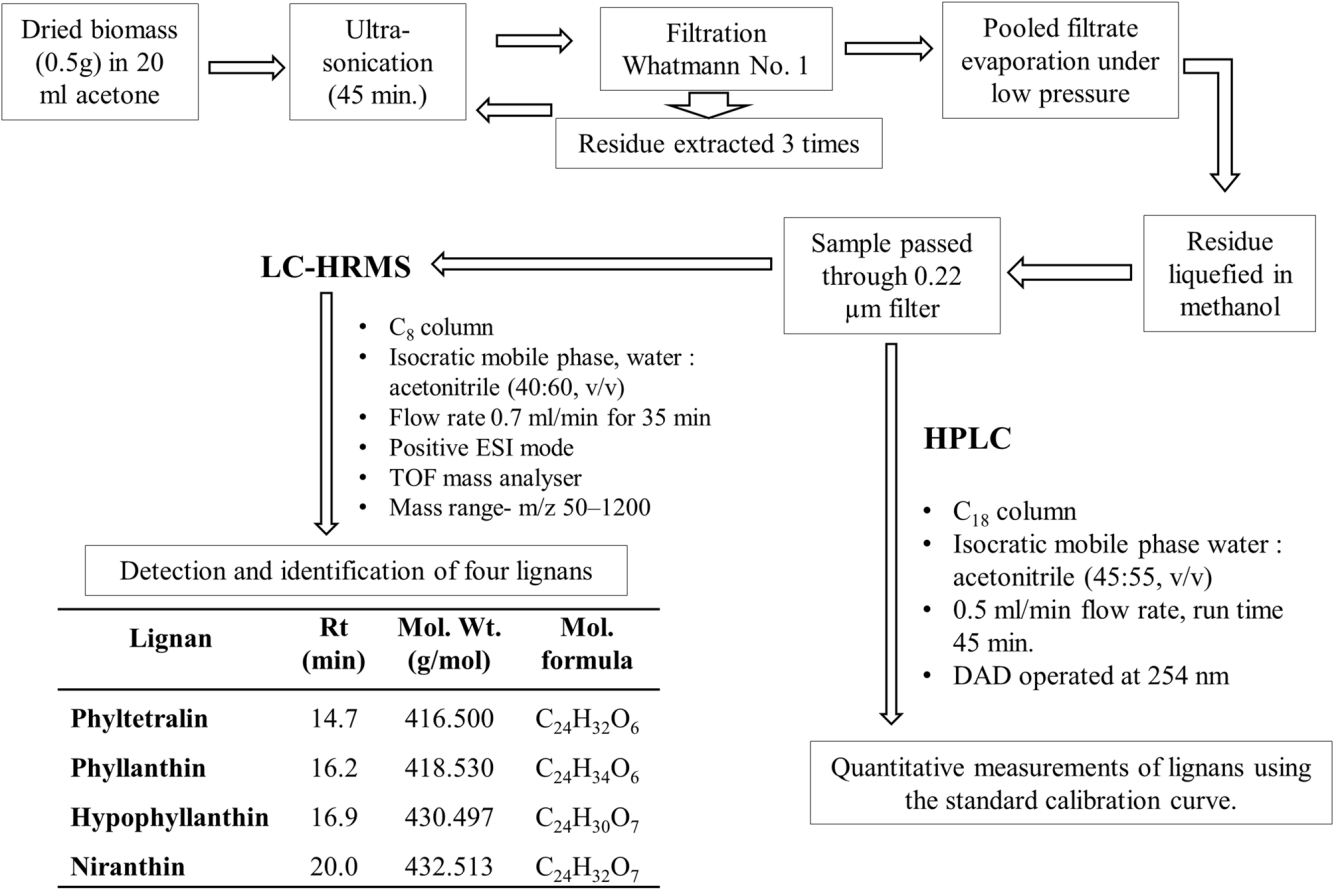
Schematic presentation of the developed protocol for detection and quantification of four lignans in naturally grown and in vitro raised biomass of *Phyllanthus tenellus*.

The resultant chromatogram consisted of several peaks in acetone extract of naturally grown as well as in vitro raised biomass of *P. tenellus*. Out of which, the peaks appeared at 14.7, 16.2, 16.9 and 20.2 min. and were detected and confirmed as phyltetralin, phyllanthin, hypophyllanthin and niranthin, respectively by comparing *Rt* and ESI mass spectra of available reference standards (Table 3, Figs. 2, 3, 4, Supplementary Fig. 1a, b). The peak appearing at 14.7 min. of phyltetralin has molecular formula as C_24_H_32_O_6_ and molecular weight (m/z 439.2002) was present with adduct ions [M + Na]. The chromatogram of standard reconfirmed the presence of phyltetralin at the same retention time as 14.7 min. with the same molecular formula and molecular weight. Therefore, the compound was assigned as phyltetralin (Figs. 2a and 3a). The peak of phyllanthin was recorded at 16.2 min. as molecular formula (C_24_H_34_O_6_) and molecular weight (m/z = 441.2167) with adduct ions [M+Na]. The signal from standard phyllanthin fits in retention time and MS spectra. Therefore, the peak was confirmed as phyllanthin (Figs. 2b and 3b). The peak of standard hypophyllanthin appeared at Rt 17.0 min. and the mass spectra showed the characteristic adduct ion at m/z 453.1798 [M+Na]. In plant extract also, corresponding mass spectra were observed with adduct ion [M+Na] m/z at 453.1811 and molecular formula (C_24_H_30_O_7_) were recorded which appeared at retention time 16.9 min. (Figs. 2c and 3c). Niranthin appeared at Rt 20.0 min. with calculated molecular mass m/z 455.1953 and molecular formula (C_24_H_32_O_7_) with adduct ion [M+Na]. Similar mass spectra were observed at retention time 20.2 min. in the chromatogram of plant extract. Therefore, the peak was identified as niranthin (Figs. 2d and 3d).

**Table 3:** Bioactive lignans detection and characterisation in *P. tenellus* by using LC-HRMS.

Type of sample	Lignans	Retention time (min)	Molecular weight (g/mol)	Experimental mass (M+Na) (m/z)	Molecular formula	Error (ppm)
Standard	Phyltetralin	14.7	416.500	439.2002	C_24_H_32_O_6_	20.30
	Phyllanthin	16.2	418.530	441.2164	C_24_H_34_O_6_	19.10
	Hypophyllanthin	16.9	430.497	453.1798	C_24_H_30_O_7_	18.90
	Niranthin	20.0	432.513	455.1953	C_24_H_32_O_7_	19.10
*P. tenellus* extract	Phyltetralin	14.7	416.500	439.2007	C_24_H_32_O_6_	19.10
	Phyllanthin	16.2	418.530	441.2167	C_24_H_34_O_6_	18.20
	Hypophyllanthin	17.0	430.497	453.1811	C_24_H_30_O_7_	16.00
	Niranthin	20.2	432.513	455.1968	C_24_H_32_O_7_	15.80

In the present investigation, total four lignans were identified in acetone extract of *P. tenellus* by using LC-HRMS. Similarly, using UPLC–ESI–MS/MS, fifty-two compounds including phyllanthin and hypophyllanthin were characterized in ethanol extract of *P. amarus*^34^. Using the same technique, there was no report on niranthin and phyltetralin. However, identification and characterization of only quercetin, ellagic acid, kaempferol and their derivatives was carried out in ethanol extract of *P. emblica, P. fraternus, P. amarus and P. niruri*^35^. The lignans phyllanthin, hypophyllanthin, niranthin and ellagic acid were identified by using LC–MS in methanol extract of *P. amarus* and *P. urinaria*^36^. Twenty phenolic compounds were recorded in UPLC–ESI–MS analysis but phyllanthin, hypophyllanthin, niranthin and phyltetralin were not recorded in methanol extract of *P. acuminatus*^37^. Using reversed phase HPLC technique, the lignans phyllanthin, hypophyllanthin, niranthin and phyltetralin were reported in *P. amarus*, *P. maderaspatensis*, *P. urinaria, P. fraternus* and *P. virgatus*^38,39^. However, only lignans niranthin, nirtetralin and hinokinin were reported in methanol extract of *P. tenellus* but not phyllanthin, hypophyllanthin, and phyltetralin^7^. To the best of our knowledge, the current study is the first report for the detection and quantification of phyllanthin, hypophyllanthin, and phyltetralin in acetone extract of *P. tenellus*. The difference in detection might be due to different solvents used for extraction and separation and detection capacity of the systems used.

### Effect of PGRs on lignans accumulation

**Figure 5:**
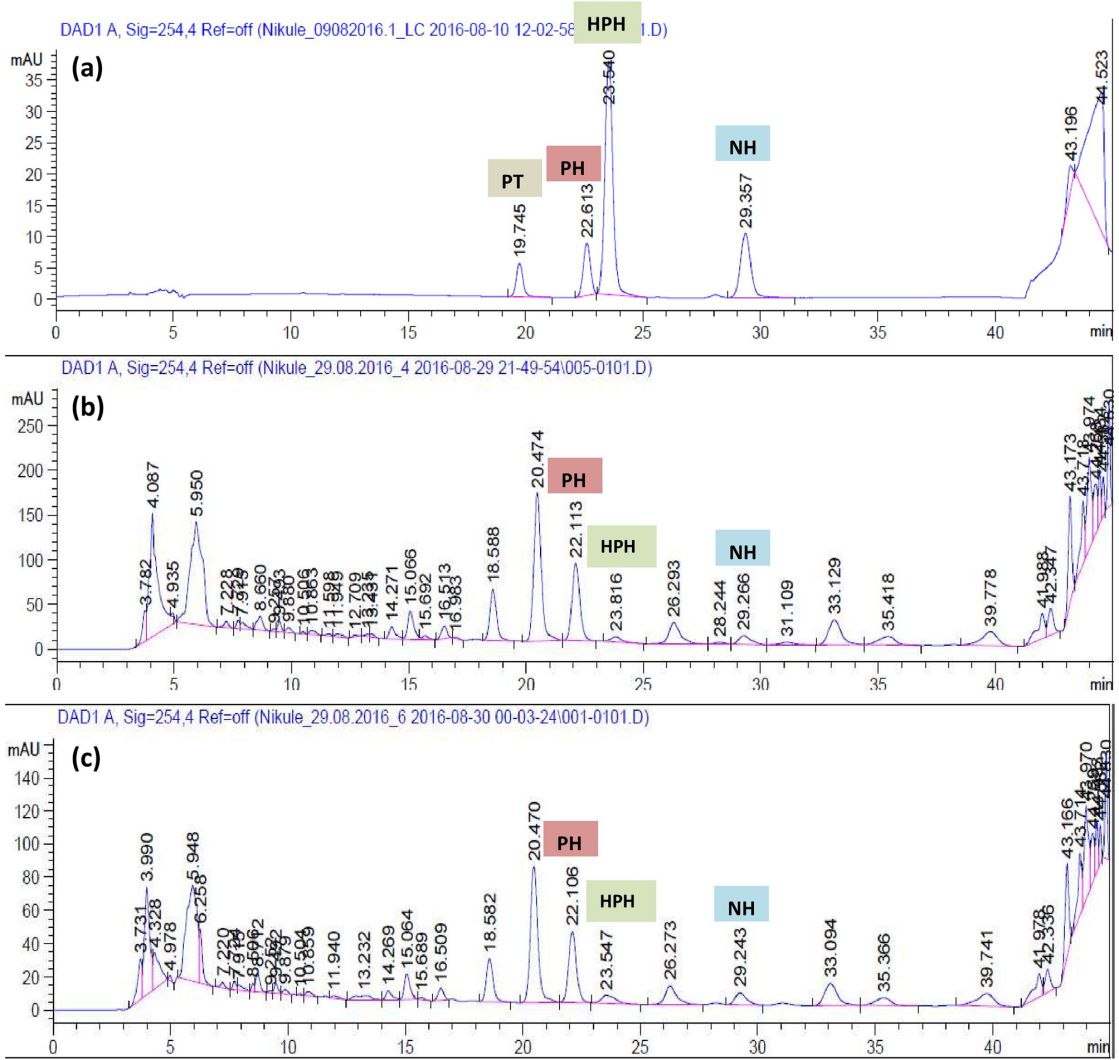
HPLC chromatograms for detection and quantification of lignans from in vitro grown plants in comparison with naturally grown plant of *P. tenellus*: (**a**) Chromatogram for standard peaks, (**b**) Chromatogram for naturally grown plant, and (**c**) Chromatogram for callus from in vitro grown plant.

**Figure 6:**
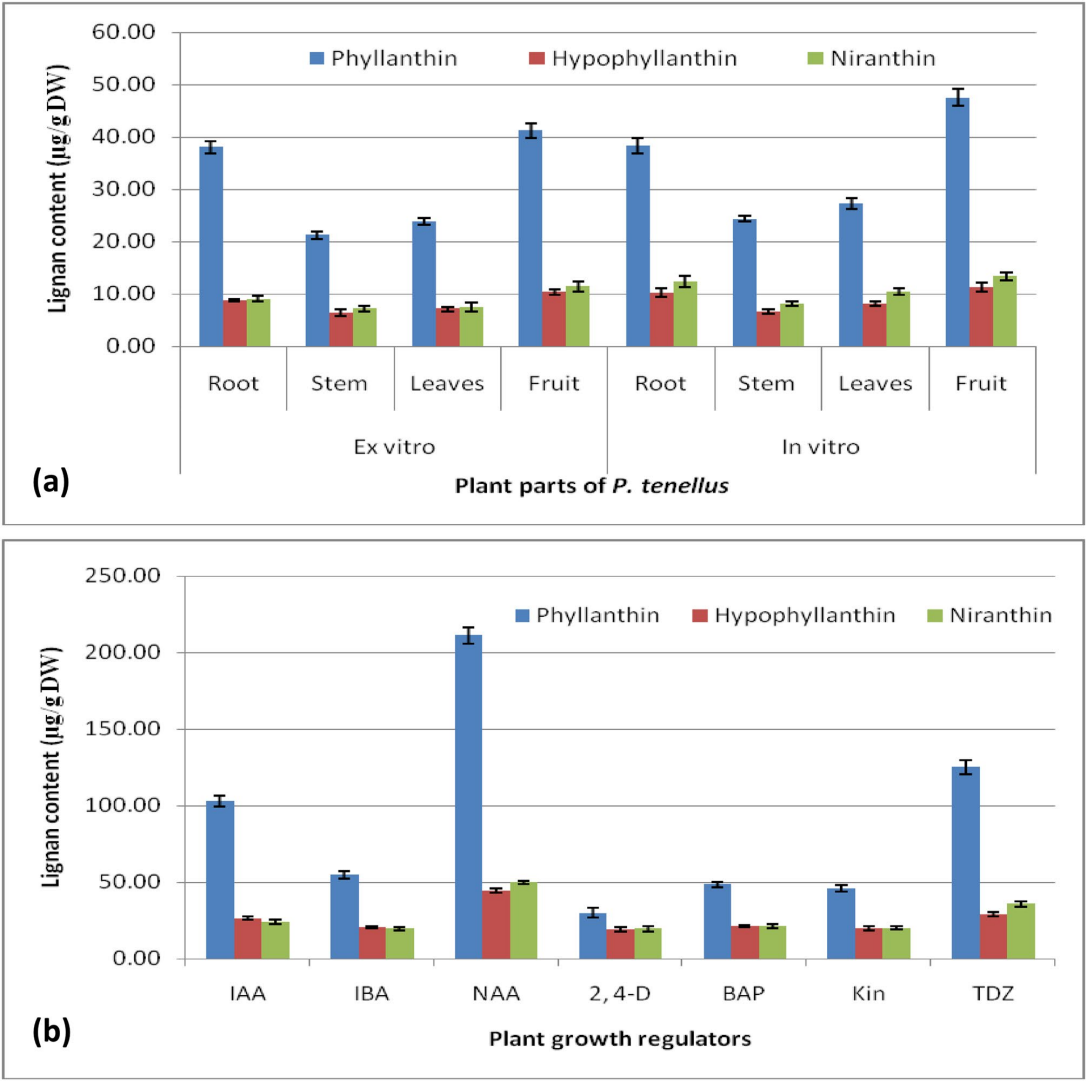
Lignan (PH, HPH and NH) content (µg/g DW ± SE) in acetone extracts from biomass of *P. tenellus*: (**a**) Plant parts of in vitro and ex-vitro grown plants, and (**b**) Effect of auxins and cytokinins on accumulation of lignan in leaf derived callus. Data represents mean ± standard error of three replicates.

Plant growth regulators play a significant role in biosynthesis of secondary metabolites in plants^40^. In the present investigation, HPLC was used to quantitative determination of lignans (Fig. 5). The obtained data showed that the lignans content was variable in *Phyllanthus tenellus *in vitro raised plantlets, field-grown plants and in callus grown on media containing different auxins (IAA, IBA, 2, 4-D, NAA) and cytokinins (TDZ, BAP, and Kin). The content of PH, HPH and NH were slightly higher in different organs of in vitro raised plantlets compared to the organs of ex-vitro grown plants (Fig. 6a, Supplementary Table 3). However, compared to field and in vitro grown plant parts, the content of lignan PH, HPH and NH was about double to triple times higher in leaf derived callus grown on different media. The content was higher in callus obtained on NAA; followed by TDZ and IAA respectively, whereas the content was low in callus produced on MS incorporated with IBA, BAP, and Kin (Fig. 6b, Supplementary Table 4). Similarly, in earlier reports, the inclusion of NAA in the medium was effective for accumulation of PH and HPH in callus of *P. amarus*^19^ and *P. urinaria*^41^ and in other plant species such as berberin in cell culture of *Thalictrum minus*^42^, catharanthine in cell culture of *Catharanthus roseus*^43^ and rutin in root cultures of *Fagopyrum esculentum*^44^. In the present study, among auxins and cytokinins used, the content of lignans was low in callus grown on medium incorporated with 2, 4-D. Similar results were reported in *P. amarus*^19,45^.

In previous studies, variable quantity of lignans PH and HPH were reported in *P. amarus* and different species of *Phyllanthus*^46–49^ (Supplementary Table 5). The maximum content of lignans PH and HPH was observed in *P. amarus* followed by *P. niruri* and next it was recorded in the current study in *P. tenellus.* PT and NH were quantified only in *P. amarus and P.niruri.* Therefore, the results of the present study have revealed that *P. tenellus* can be one of the best sources of bioactive lignans PH, HPH and NH. The differences in metabolite content in source material may be due to the influence of various factors like ecological conditions, age of the plant parts, drying methods, extraction methods, extraction solvents, and type of analytical techniques used.

## Conclusion

The present investigations are the first report on detection and quantification of bioactive lignans phyllanthin, hypophyllanthin, niranthin and phyltetralin in *Phyllanthus tenellus.* The herb may emerge as one of the best source of bioactive lignans. The in vitro propagation protocol would be useful for the application of biotechnological approaches for improvement in phyto-pharming. This study revealed that lignan compounds accumulation was influenced by the in vivo and in vitro conditions, type of auxins and cytokinins and their concentration in the nutrient medium. The content of lignans in callus culture can be significantly augmented by the presence of NAA in the nutrient medium. Further, these techniques can be used to scale up the process of lignan production at industrial level round the year. The results tend to support the use of *Phyllanthus tenellus* in country wise developed traditional medicinal systems to cure diseases and disorders.

## Supplementary information

Supplementary information
