# Supplementary material for: In-vitro propagation, callus culture and bioactive lignan production in Phyllanthus tenellus Roxb: a new source of phyllanthin, hypophyllanthin and phyltetralin
Source: Sci Rep. 2020 Jun 30;10:10668. doi: 10.1038/s41598-020-67637-8 (PMC7327055; doi:10.1038/s41598-020-67637-8)
Supplement: Supplementary file 1 — Supplementary information [file 41598_2020_67637_MOESM1_ESM.pdf]

***In-vitro* propagation, callus culture and bioactive lignan production in *Phyllanthus tenellus* Roxb: A new source of phyllanthin, hypophyllanthin and phyltetralin**

Harichandra A. Nikule[1,2], Kirti M. Nitnaware[3], Mahadev R. Chambhare[1], Nitin S. Kadam[1,4], Mahesh Y. Borde[1], Tukaram D. Nikam[1]*

[1]Department of Botany, Savitribai Phule Pune University, Pune 411 007, India.

[2]Central Instrumentation Facility, Savitribai Phule Pune University, Pune 411 007, India.

[3]Department of Botany, Hutatma Rajguru Mahavidyalaya, Rajgurunagar-410 505, Dist. Pune, India

[4]Design Innovation Centre, Department of Chemistry, Savitribai Phule Pune University, Pune 411 007, India.

*Corresponding author

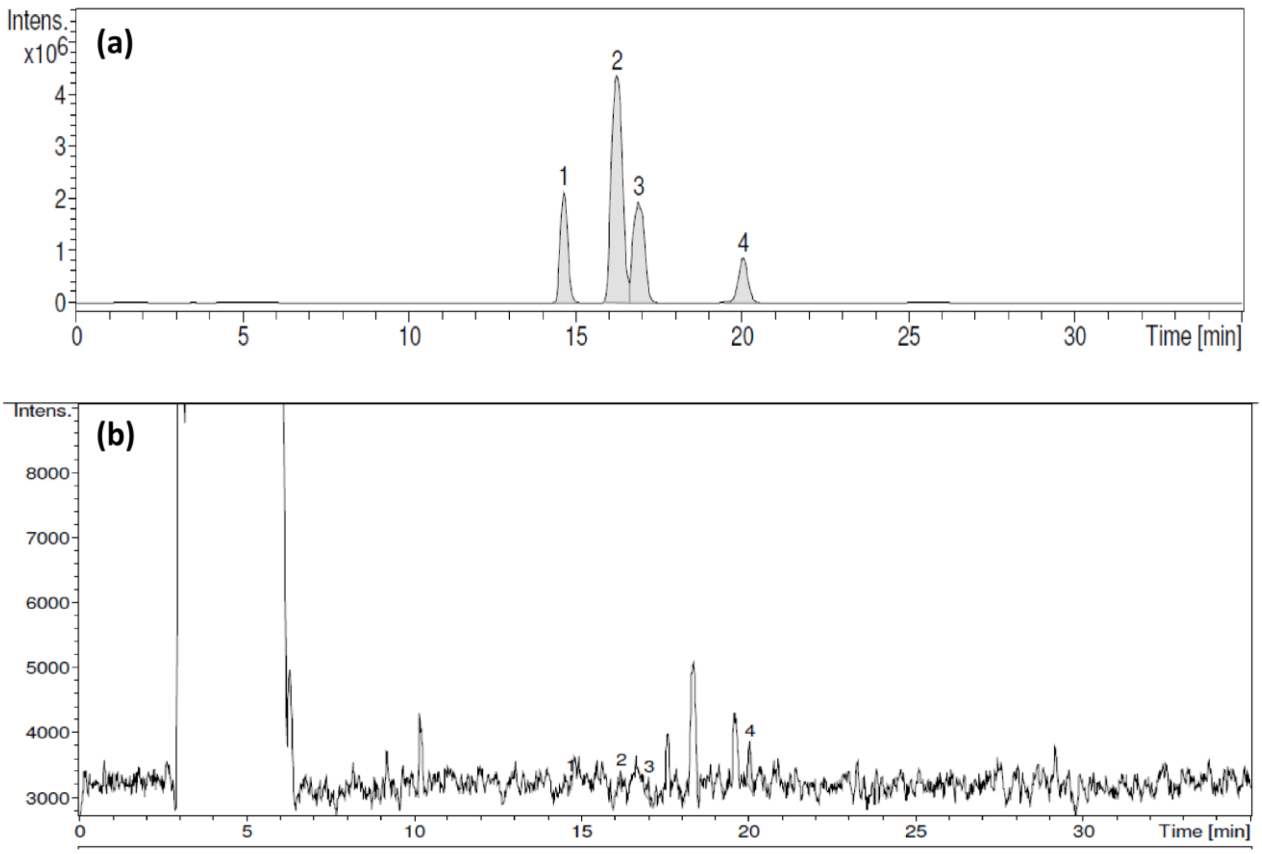

Supplementary figure 1. (**a)** LC-MS base peak chromatograms (BPC): (a) BPC of reference standards: Phyltetralin (1), Phyllanthin (2), Hypophyllanthin (3), Niranthin (4). (**b)** Base peak chromatograms (BPCs) of acetone extract of *in vitro* grown *P. tenellus* extracts; Phyltetralin (1), Phyllanthin (2), Hypophyllanthin (3), Niranthin (4).

PGRs	Conc. (mg/l)	Explants			
		Node		Leaf	
		Response of Explant for root formation % ± SE	No. of Roots/ explant ± SE	Response of Explant for root formation % ± SE	No. of Roots/ explant ± SE
Control	0	86.03 ± 0.18 ^{l}	4.07 ± 0.09 ^{kl}	0.00 ± 0.00 ^{n}	0.00 ± 0.00 ^{s}
IAA	0.5	97.63 ± 0.48 ^{b}	11.67 ± 0.24 ^{d}	90.03 ± 0.64 ^{ijk}	8.87 ± 0.18 ^{g}
	1	100.00 ± 0.00 ^{a}	17.83 ± 0.34 ^{b}	92.57 ± 0.35 ^{d}	9.33 ± 0.15 ^{f}
	2	100.00 ± 0.00 ^{a}	18.53 ± 0.18 ^{a}	94.23 ± 0.23 ^{c}	14.53 ± 0.18 ^{b}
	3	97.20 ± 0.21 ^{b}	15.80 ± 0.12 ^{c}	93.53 ± 0.30 ^{c}	10.30 ± 0.12 ^{e}
IBA	0.5	86.57 ± 0.20 ^{l}	4.60 ± 0.12 ^{ij}	86.60 ± 0.23 ^{m}	4.70 ± 0.12 ^{m}
	1	87.27 ± 0.18 ^{k}	4.80 ± 0.12 ^{i}	88.33 ± 0.13 ^{l}	6.20 ± 0.12 ^{k}
	2	89.57 ± 0.22 ^{j}	4.83 ± 0.15 ^{i}	92.27 ± 0.20 ^{de}	8.80 ± 0.12 ^{g}
	3	88.37 ± 0.12 ^{h}	4.30 ± 0.12 ^{ijk}	91.80 ± 0.15 ^{def}	7.30 ± 0.12 ^{i}
NAA	0.5	90.63 ± 0.38 ^{i}	4.83 ± 0.09 ^{i}	91.63 ± 0.24 ^{efg}	8.40 ± 0.12 ^{h}
	1	94.53 ± 0.18 ^{e*}	6.20 ± 0.12 ^{h*}	97.30 ± 0.12 ^{a*}	13.47 ± 0.09 ^{c*}
	2	96.53 ± 0.24 ^{c**}	6.40 ± 0.12 ^{h**}	97.37 ± 0.12 ^{a**}	15.20 ± 0.12 ^{a**}
	3	92.53 ± 0.18 ^{g**}	4.73 ± 0.07 ^{i**}	96.40 ± 0.12 ^{b***}	12.30 ± 0.12 ^{d***}

Supplementary table 1. Effect of different concentrations of auxins (IAA, IBA and NAA) on root formation in *Phyllanthus tenellus* Roxb. Data represent the means of three replicates with standard error (SE). Values sharing the different alphabets are statistically different from each other at $P < 0.05$. *Callus induction; **Callus induction and proliferation; ***Extensive Callus Proliferation.

PGRs	Conc. (mg/l)	Explant					
		Node			Leaf		
		Nature of callus	Fresh weight (g) ± SE	Dry weight (mg) ± SE	Nature of callus	Fresh weight (g) ± SE	Dry weight (mg) ± SE
Control	00	N	N	N	N	N	N
2, 4-D	0.5	GYF	1.24 ± 0.01^{efg}	192.20 ± 0.89^{g}	GWF	1.21 ± 0.01^{l}	219.13 ± 1.77^{gh}
	1	GYF	1.34 ± 0.01^{b}	203.93 ± 0.72^{f}	GWF	1.84 ± 0.01^{cd}	244.57 ± 9.48^{b}
	2	GYF	1.53 ± 0.01^{a}	228.13 ± 1.07^{a}	GWF	1.93 ± 0.01^{a}	259.47 ± 1.16^{a}
	3	GYF	1.35 ± 0.02^{b}	213.97 ± 0.68^{cd}	GWF	1.78 ± 0.01^{de}	231.47 ± 1.18^{de}
2, 4-D + BAP	0.5	GC	1.27 ± 0.01^{de}	213.17 ± 0.72^{cd}	GF	1.42 ± 0.02^{g}	223.40 ± 1.18^{fg}
	1	GC	1.11 ± 0.01^{h}	203.60 ± 0.67^{f}	GF	1.26 ± 0.02^{jk}	211.83 ± 2.02^{i}
	2	GC	0.79 ± 0.01^{j}	192.27 ± 0.91^{g}	GF	0.92 ± 0.01^{p}	195.73 ± 1.52^{j}
	3	GC	0.76 ± 0.02^{k}	179.73 ± 0.79^{i}	GF	0.86 ± 0.01^{q}	171.47 ± 1.19^{l}
2, 4-D + KIN	0.5	YGC	1.35 ± 0.02^{b}	218.43 ± 1.16^{b}	GYF	1.31 ± 0.01^{hi}	248.27 ± 1.82^{b}
	1	YGC	1.24 ± 0.01^{ef}	206.30 ± 0.47^{e}	GYF	1.28 ± 0.01^{ij}	232.90 ± 1.12^{de}
	2	YGC	1.20 ± 0.01^{g}	179.80 ± 0.85^{i}	GYF	1.12 ± 0.01^{n}	213.37 ± 0.65^{hi}
	3	YGC	1.11 ± 0.01^{h}	172.73 ± 0.91^{j}	GYF	1.02 ± 0.02^{o}	193.97 ± 0.92^{jk}
2, 4-D + TDZ	0.5	GWC	1.31 ± 0.01^{bc}	211.33 ± 0.60^{d}	GWF	1.84 ± 0.02^{cd}	241.40 ± 1.88^{bc}
	1	GWC	1.29 ± 0.02^{cd}	202.03 ± 1.05^{f}	GWF	1.78 ± 0.01^{de}	234.53 ± 1.16^{cde}
	2	GWC	1.09 ± 0.01^{h}	186.23 ± 0.58^{h}	GWF	1.73 ± 0.01^{f}	213.50 ± 1.04^{hi}
	3	GWC	1.02 ± 0.02^{i}	170.63 ± 1.11^{j}	GWF	1.32 ± 0.01^{h}	189.30 ± 1.24^{jk}

Supplementary table 2. Effect of different concentrations of 2, 4-D alone and in combination with cytokinins on growth of callus in *Phyllanthus tenellus* Roxb. Data represent the means of three replicates with standard error (SE). Values sharing the different alphabets are statistically different from each other at $P < 0.05$. GYF, Green Yellowish Friable; GC, Green Compact; YGC, Yellow Greenish Compact; GWC, Green Whitish Compact; GWF, Greenish White Friable; GF, Green Friable.

Type of plant	Plant part	Phyllanthin (µg/g dry weight)	Hypophyllanthin (µg/g dry weight)	Niranthin (µg/g dry weight)
Ex vitro	Root	38.0±1.1	8.8±0.2	9.0±0.5
	Stem	21.3±0.7	6.4±0.7	7.2±0.6
	Leaves	23.8±0.6	7.1±0.4	7.5±0.8
	Fruit	41.3±1.4	10.3±0.6	11.4±1.0
In vitro	Root	38.3±1.5	10.2±0.8	12.4±1.0
	Stem	24.4±0.5	6.6±0.5	8.1±0.4
	Leaves	27.3±1.0	8.1±0.5	10.5±0.6
	Fruit	47.5±1.6	11.3±0.8	13.4±0.8

Supplementary table 3. Lignan content in different parts of *Phyllanthus tenellus*. Data represent the means of three replicates with standard error (SE). Values sharing the different alphabets are statistically different from each other at $P < 0.05$.

PGRs	Phyllanthin (µg/g DW)	Hypophyllanthin (µg/g DW)	Niranthin (µg/g DW)
IAA	103.0±3.5	26.3±1.0	24.2±1.2
IBA	54.6±2.6	20.2±0.7	19.5±1.4
NAA	211.0±5.6	44.3±1.5	49.9±1.0
2, 4-D	29.8±3.3	19.1±1.1	19.5±1.5
BAP	48.3±2.0	21.3±0.8	21.4±1.3
Kin	46.0±2.1	19.8±1.3	20.0±1.0
TDZ	125.2±4.5	28.8±1.6	35.6±1.7

Supplementary table 4. Effect of auxins and cytokinins on lignan content in callus of *Phyllanthus tenellus*. Data represent the means of three replicates with standard error (SE). Values sharing the different alphabets are statistically different from each other at $P < 0.05$.

Sr. No.	*Phyllanthus spp.*	Lignan content (µg/g)				Reference
		PH	HPH	NH	PT	
1	*P. amarus*	8120	NR	NR	NR	[46]
		5300	3200	NR	NR	[47]
		1270	510	2010	NR	[48]
		258.3	128.3	NR	NR	[19]
		170	140	NR	NR	[49]
		14.3	11.06	10.59	NR	[36]
2	*P. niruri*	7540	3560	6860	1080	[20]
3	*P. tenellus*	211.05	44.35	49.93	NR	Present work
4	*P. fraternus*	200	NR	NR	NR	[47]
5	*P. virgatus*	200	NR	NR	NR	[47]
6	*P. maderaspatensis*	100	NR	NR	NR	[47]
7	*P. urinaria*	0.115	0.737	0.056	NR	[36]

Supplementary table 5. Lignan content reported from various *Phyllanthus spp.* NR, Not Reported.
